# Theoretical Compartment Modeling of DCE-MRI Data Based on the Transport across Physiological Barriers in the Brain

**DOI:** 10.1155/2012/482565

**Published:** 2012-05-14

**Authors:** Laura Fanea, Leontin I. David, Andrei Lebovici, Francesca Carbone, Silviu A. Sfrangeu

**Affiliations:** ^1^Department of Biomedical Physics, Physics Faculty, Babes-Bolyai University, No. 1 M. Kogalniceanu Street, 400084 Cluj-Napoca, Romania; ^2^Department of Radiology, Cluj County Emergency Hospital, No. 3-5, Clinicilor Street, 400006 Cluj-Napoca, Romania; ^3^Faculty of Pharmacy, University of Salerno, Via Ponte Don Melilo, 84084 Fisciano, Italy

## Abstract

Neurological disorders represent major causes of lost years of healthy life and mortality worldwide. Development of their quantitative interdisciplinary *in vivo* evaluation is required. Compartment modeling (CM) of brain data acquired *in vivo* using magnetic resonance imaging techniques with clinically available contrast agents can be performed to quantitatively assess brain perfusion. Transport of ^1^H spins in water molecules across physiological compartmental brain barriers in three different pools was mathematically modeled and theoretically evaluated in this paper and the corresponding theoretical compartment modeling of dynamic contrast enhanced magnetic resonance imaging (DCE-MRI) data was analyzed. The pools considered were blood, tissue, and cerebrospinal fluid (CSF). The blood and CSF data were mathematically modeled assuming continuous flow of the ^1^H spins in these pools. Tissue data was modeled using three CMs. Results in this paper show that transport across physiological brain barriers such as the blood to brain barrier, the extracellular space to the intracellular space barrier, or the blood to CSF barrier can be evaluated quantitatively. Statistical evaluations of this quantitative information may be performed to assess tissue perfusion, barriers' integrity, and CSF flow *in vivo* in the normal or disease-affected brain or to assess response to therapy.

## 1. Introduction

Neurological disorders are diseases of the central and peripheral nervous system affecting approximately one billion people worldwide irrespective of age, sex, education, or income. The most frequent neurological disorders at present are migraines, followed by neurological problems caused by cerebrovascular disease, epilepsy, Alzheimer's disease, and so forth [1]. Hydrocephalus is especially painful, representing the leading cause for brain surgery in children in the United States of America [2]. Neuropsychiatric disorders and injuries, in particular, represent major causes of lost years of healthy life and are significantly underestimated when measured by mortality alone [1].

Impact of neurological disorders is expected to increase, for example, the number of people affected by Alzheimer's disease and other dementia conditions is expected to double every twenty years. Almost seven million people die every year as a result of neurological disorders, the most affected being people in the lower middle income category. Investigation of neurological disorders increaseas in importance due to prolonged ageing also [1].

Neuroscientifically relevant observations were recorded since 4000 BC and they contributed to the development of neurosciences [3]. During the previous two centuries, brain research belonged to many different areas that differed in methodology and targets: the morphological, the physiological, and the psychological. The latter used to consider the brain as a black box where only the input and output were known but not at all the neuronal components and the way they interact with each other. More recently, scientific and technological research, from molecular to behavioural levels, have been carried out but they have not been developed in a really interdisciplinary way [1].

Kinetics of an agent through physiological barriers, including physiological brain barriers, can be quantitatively evaluated *in vivo, *using exogenous or endogenous magnetic resonance imaging (MRI) techniques and compartment modeling (CM) of the MRI data [4–6]. Theoretical description of two and three CMs of endogenous (without contrast agents for MRI, the ASL techniques) and exogenous (with extra- or intracellular contrast agents for MRI) perfusion MRI techniques have been described [4–6]. The signal-to-noise ratio of the data acquired using endogenous perfusion MRI techniques is still much lower than that of the data obtained with exogenous perfusion MRI techniques. Intracellular contrast agents present at the moment are only used in clinical trials. Perfusion MRI techniques using extracellular contrast agents (the DCE-MRI techniques) are less invasive and they are routinely used in clinical MRI, including cerebral MRI.

Mathematical modeling of data acquired using exogenous perfusion MRI techniques with intracellular contrast agents (the ssCE techniques) which might become mathematically very complex since there will be more mechanisms of relaxation time modifications in each compartment of a voxel (i.e., volumetric image element) to be considered. This will lead to more complex equations to be solved and would introduce more parameters to be estimated when fitting the MRI data to the mathematical model developed.

Mathematical modeling of the brain data acquired *in vivo* using exogenous perfusion MRI techniques with extracellular contrast agents is easier since the signal-to-noise ratio of the MRI data is the highest and the contrast agent does not penetrate in the extracellular space (EES) through the intact blood-to-brain barrier (BBB). Increase in the signal-to-noise ratio of the DCE-MRI has recently been obtained using a slow infusion technique of Gd-based extracellular clinically available contrast agents [7]. This infusion technique allowed a much easier mathematical modeling of the mouse brain DCE-MRI data. Slow infusion techniques can also be used clinically. Yankeelov et al. [8] used a slow infusion technique of Gd-based extracellular contrast agents to quantitatively evaluate DCE-MRI data of the breast.

The mathematical modeling of the perfusion MRI data in general and DCE-MRI data in particular, based on the physiological compartmentalization of voxels, gives more complex and realistic information on the kinetics of agents through barriers between physiological compartments. For brain and brain conditions, this might allow quantitative assessment of the BBB, blood-to-CSF barrier (B-CSF-B), or that of the output to input flow (IOF) imbalance of ^1^H spins in water molecules through the CSF spaces.

Theoretical description of the physiological compartmentalization of tissue and CSF voxels and the mathematical modeling of the blood, tissue, and CSF data acquired using DCE-MRI and a slow infusion technique of Gd-based contrast agents is presented in this paper. Kinetics of ^1^H spins in water molecules present in two different phases (with unaffected and shortened spin-lattice relaxation time, *T_1_*) in the three pools assumed (blood, tissue, and CSF) was quantitatively evaluated in this study. The relative volumes of the physiological compartments of voxels situated in the tissue and CSF regions can also be estimated using this physiological CM of the DCE-MRI brain data.

Results in this study show that quantitative information can be extracted from brain data acquired *in vivo* using DCE-MRI techniques. Integrity of physiological barriers in the brain, IOF imbalances in CSF spaces, and relative volumes of physiological compartments can be assessed using this quantitative information.

Future possible statistical experimental studies using these CMs might provide quantitative information on the BBB and B-CSF-B integrity and/or shrinkage of the brain tissue in the diseased brain, for example. Potential of these CMs for the *in vivo* quantitative monitoring of neurological disorders, therapies, or normal brain function can also be assessed.

## 2. Mathematical Model

The ^1^H nuclei relevant for DCE-MRI images in the blood, tissue, and CSF pools exist in two different phases: one with unaffected longitudinal relaxation time *T_1_* and one with* T_1_* shortened. The relaxation time* T_1_* is shortened due to the spin-lattice interactions between the ^1^H nuclei in water molecules with the paramagnetic Gd ions in the exogenous clinically available contrast agents for MRI. Transport of the ^1^H nuclei in these two aqueous phases across the barriers between the compartments of the pools evaluated was mathematically modeled.

### 2.1. Transport in the Blood Pool

Infusion of the agent in the blood pool is continuous and, therefore, the ^1^H spins in the two phases flow continuously in this pool [9–11] at a concentration rate *C*_IN_ and leave the pool at a rate *k*_OUT_, their kinetics being described by the following arterial input function, AIF:

(1) $$\text{AIF}\left(t\right)=C_{\text{IN}}te^{-tk_{\text{OUT}}}.$$

The AIF in (1) refers to the time points before and during injection. No more contrast agents will enter the blood compartment after the end of the slow infusion of the contrast agent, only the elimination mechanisms will take place during this period of time.

Concentration of the ^1^H spins in water molecules in the mixed phases in the blood, *C*_BLOOD_, depends on the hematocrit, Hct, level and is given by the arterial input function multiplied by the (1-Hct) factor.

### 2.2. Tissue Perfusion

**Figure 1:**
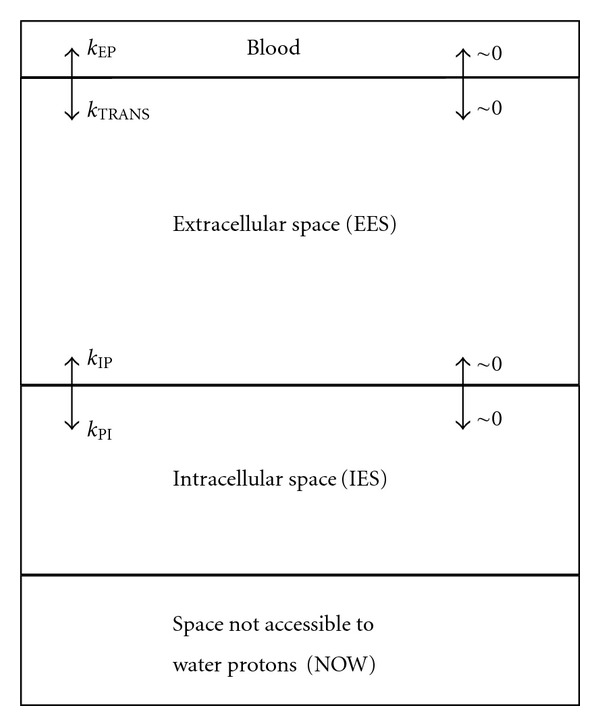
Physiological compartmentalization of a tissue voxel in the region of white or gray matter.

Each voxel corresponding to white or gray matter regions was compartmentalized as shown in Figure 1. The four physiological compartments of a tissue voxel are blood (accessible to ^1^H spins in water molecules), extracellular space (EES—accessible to ^1^H spins in water molecules), intracellular space (IES—accessible to ^1^H spins in water molecules), and a space not accessible to water (NOW—not accessible to ^1^H spins in water molecules). The bidirectional transport of the ^1^H spins across the blood to EES and the EES to IES barriers are represented with arrows. Rates of ^1^H spins with unaffected *T_1_* can be neglected (~0) compared to that of ^1^H spins with short *T_1_*. The fast and slow exchange of the ^1^H spins in water molecules between these compartments is bidirectional. These exchanges together with accumulation of the ^1^H spins in the two phases over time in a compartment produce dynamic changes of the amplitude of the corresponding nuclear magnetic resonance (NMR) signals.

The four tissue compartments in Figure 1 are the blood, the EES, the intracellular space (IES), and a space not accessible to ^1^H nuclei in water molecules (NOW). The relative volumes of these compartments: *v*_BLOOD_, *v*_EES_, *v*_IES_, and *v*_NOW_ fulfill the condition:

(2) $$v_{\text{BLOOD}}+v_{\text{EES}}+v_{\text{IES}}+v_{\text{NOW}}=1.$$

Transport of the ^1^H spins in water molecules across barriers is bidirectional. Its mechanisms are similar to action potential transport across cellular membranes due to the concentration gradient of the ^1^H spins in a phase in a compartment.

The concentration of the ^1^H spins producing changes of the NMR signal in a tissue, *C*_TISSUE_, depends on the concentration of the ^1^H nuclei producing changes of the NMR signals in each of these compartments: *C*_BLOOD_, *C*_EES_ and *C*_IES_

(3) $$C_{\text{TISSUE}}\left(t\right)=v_{\text{BLOOD}}C_{\text{BLOOD}}\left(t\right)+v_{\text{EES}}C_{\text{EES}}\left(t\right)+v_{\text{IES}}C_{\text{IES}}\left(t\right).$$

The fast and slow transport of the ^1^H spins in the two phases across the blood to EES and the EES to IES barriers also contributes to the NMR signal intensity changes over time.

#### 2.2.1. Bidirectional Transport across the Blood to EES Barrier

Concentration of the ^1^H spins producing changes of the MRI signal intensity in a voxel due to its transport from the blood into the EES compartment can be determined by solving the Kety-Schmidt equation (4) for each phase of the ^1^H nuclei in water molecules.

Gd-based extracellular contrast agents do not penetrate into the EES space while the BBB is intact and the ^1^H spins with shortened *T_1_* are moving much slower compared with those with unaffected *T_1_* [4, 5]. Contribution of the ^1^H spins with unaffected *T_1_* can, therefore, be neglected and the Kety-Schmidt equation that needs to be solved is given below in (4).

The rate of the concentration variation of the ^1^H spins in water molecules in the EES compartment, “*dC*_EES_/*dt*” increases due to the transport of the ^1^H nuclei with shortened *T_1_* from the blood into the EES compartment at a rate *k*_TRANS_ and decreases due to these spins reentering the blood compartment at a rate *k*_*EP*⁡_:

(4) $$\frac{dC_{\text{EES}}\left(t\right)}{dt}+k_{\mathrm{EP}}C_{\text{EES}}\left(t\right)=k_{\text{TRANS}}C_{\text{BLOOD}}\left(t\right).$$

All initial concentrations (at *t* = 0) are zero.

Equation (4) is a linear nonhomogeneous first order differential equation whose general solution is given in the Appendix.

#### 2.2.2. Bidirectional Transport across the EES to IES Barrier

Extracellular contrast agents do not enter the IES compartment and contribution of ^1^H spins with unaffected *T_1_* to the signal intensity change can again be neglected on the assumption made in the previous subsection. The rate of the concentration variation of the ^1^H spins in water molecules in the IES space, *dC*_IES_/*dt*, increases due to the transport of the ^1^H nuclei with shortened *T_1_* from the EES into the IES compartment at a rate *k*_PI_ and decreases due to these spins reentering the EES compartment at a rate *k*_IP_:

(5) $$\frac{dC_{\text{IES}}\left(t\right)}{dt}+k_{\text{IP}}C_{\text{IES}}\left(t\right)=k_{\text{PI}}C_{\text{EES}}\left(t\right),$$

with *C*_EES_(*t*) obtained by solving (4). Solution to this equation is also given in the Appendix.

### 2.3. Flow in the CSF Spaces

**Figure 2:**
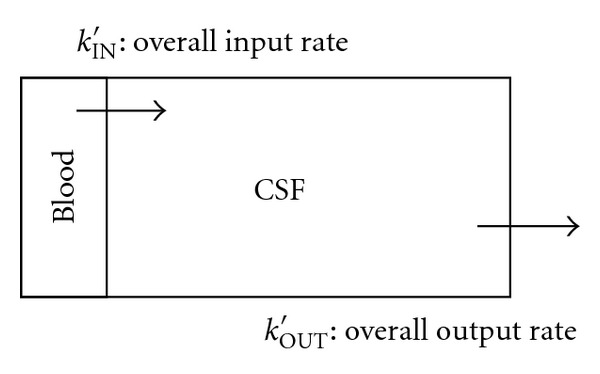
Physiological compartmentalization of a CSF voxel.

CSF is formed by three different important mechanisms and flows several regions in the brain [12]. Only the main mechanism of CSF formation, that of the blood filtration, was considered in this study. Kinetics of the ^1^H spins producing changes of the MRI signal intensity in CSF spaces will, therefore, be different compared to that in the tissue. Each voxel in a CSF region can be compartmentalized in two spaces [13] as described in Figure 2. The two physiological compartments of a CSF voxel are blood and CSF, both accessible to ^1^H nuclei in water molecules. ^1^H nuclei in water molecules in the blood are filtrated in a CSF region at an overall rate *k*_IN_′ and evacuate the space at an overall rate *k*_OUT_′. The fluid flowing through, accumulated in the space, represents the CSF. The arrows represent the unidirectional transport of ^1^H nuclei in water molecules across the blood to CSF barrier.

There are two pools containing ^1^H nuclei in water molecules in a CSF voxel: the blood and the CSF pool. The relative volumes of these two compartments are: *v*_BLOOD_, the relative volume of the blood pool, and *v*_CSF_, the relative volume of the CSF pool:

(6) $$v_{\text{BLOOD}}+v_{\text{CSF}}=1.$$

Flow of the mixed phases ^1^H spins in water molecules in a CSF space is continuous and it will be defined by a CSF input function (CSFIF), similar to the AIF. The spins enter a CSF space at a rate *k*_IN_′ and leave the space at a rate *k*_OUT_′

(7) $$\text{CSFIF}\left(t\right)=k_{\text{IN}}^{\prime }te^{-k_{\text{OUT}}^{\prime }t}.$$

The disturbance of the input and output flow mechanisms through the B-CSF-B barrier together with the predominance of the disturbance of a mechanism can be quantitatively assessed by the output to input flow, IOF. The output to input flow, IOF, represents the mean relative normalized rate of ^1^H nuclei in water molecules in a CSF space. The mean input/output rates of ^1^H nuclei in water molecules flowing in a CSF space are calculated for each disease stage (e.g., control, mild, and severe). Normalization of each mean input/output rate in a CSF space at a disease stage is performed against the corresponding control (normal brain) input/output mean rate in that CSF space:

(8) $$\text{IOF}=\frac{{{\overset{̅}{k}}_{\text{IN}}^{\prime }|}_{\text{NORMALIZED}}}{{{\overset{̅}{k}}_{\text{OUT}}^{\prime }|}_{\text{NORMALIZED}}}.$$

## 3. Results and Discussion

Similar blood concentration curves and AIFs were obtained for the mouse [7] and rat [13] brain DCE-MRI data. The overall input and output rates in the blood pool of the rat brains evaluated showed that the most rapid evacuation of the contrast agent from the blood pool took place at the mild stages of communicating hydrocephalus (C-HC), compared to the normal and severely C-HC-affected rat brains [13]. The values of the overall input rates in the blood pool ranged from 0.0427 to 0.0956 mM/min, while that of the overall output rates in the blood pool ranged from 0.0281 and 0.0344 1/min [13].

Transport rates between the blood compartment and the EES compartment in the tissue pool ranging between 0 and 0.0005 1/min were calculated using this compartment modeling applied to DCE-MRI images of normal and hydrocephalic rat brains [13]. The changes of the NMR signal intensities in the brain regions characterized by null *k*_TRANS_ values were produced only by the ^1^H nuclei in water molecules in the blood compartment in the tissue pool evaluated.The zero values of these rates also show that resolution and signal-to-noise ratio of the DCE-MRI technique evaluated need to be increased to detect transport rate values as small as 0.0001 1/min. All *k*_TRANS_ values calculated showed that, as opposed to normal pressure hydrocephalus [14], the BBB is intact in C-HC.

For fractional volumes of the EES compartment of 20%, the fractional volumes calculated for the blood compartment in the tissue pool (cortex and thalamus—left and right hemispheres) ranged between 0.05 and 7.98%, while the fractional volumes of the IES compartment ranged between 72 and 77% [13].

The IOF values calculated for the CSF pool clearly showed that these are the most sensitive indicators of the mechanisms of the dynamic disturbances in C-HC. The flow disturbances were more pronounced in the aqueduct (IOF = 2.49) and in the IVth ventricle (IOF = 1.96) in the mild stage of the C-HC. The IOF values calculated (IOF > 1) in the lateral ventricle (mild and severe stages of C-HC), aqueduct (mild and severe C-HC stages) and the IVth ventricle (mild C-HC stage) were larger than the corresponding IOF values calculated (IOF = 1) in the normal brain. The most pronounced increases of the IOF values were detected for the mild C-HC stages in all CSF spaces analyzed. In the IVth ventricle, the IOF value increased (IOF = 1.96) for the mild stage of C-HC and decreased below the corresponding IOF (1/IOF = 1.7) for the severe stage of C-HC. The IOF values calculated in the lateral ventricle and aqueduct increased relatively to the corresponding IOF values of the normal brain, and although they decreased in the severe C-HC stage relative to the mild stage, they remained larger than the corresponding IOF values calculated for the normal brain.

The increases of the IOF values can be correlated with decreases of the overall output rates relative to the corresponding input rates in a CSF space at a disease stage. More ^1^H spins with decreased *T_1_* will accumulate at the level of a CSF space compared to the normal brain (control stage), producing volume dilatations of these spaces compared to the corresponding spaces of the normal brain. These dilatations reduced in the lateral ventricle and aqueduct, IOF decreased with disease severity from 1.87 to 1.55 and from 2.49 to 1.4. In the IVth ventricle, IOF increased in the mild C-HC stage (IOF = 1.96) and decreased in the severe stage (1/IOF = 1.7). More ^1^H spins with decreased* T_1_* will accumulate in the CSF spaces evaluated in the mild C-HC stage (the aqueduct and the lateral and the IVth ventricles) and in the severe C-HC stage (aqueduct and lateral ventricle) compared to the normal brain (control stage). Many more ^1^H spins with decreased* T_1_* will evacuate the IVth ventricle in the severe stage of C-HC compared to the normal brain (control stage). This massive evacuation at the level of the IVth ventricle in the severe C-HC stage indicates the disruption of the B-CSF-B at this level of the CSF space in the severe C-HC stage. The calculated IOF value at the level of the IVth ventricle in the severe C-HC stage indicates the disruption of the normal evacuation mechanisms of the ^1^H spins with decreased *T_1_* at this level of the CSF and for this C-HC stage. The evacuation, in this situation, is made as if no B-CSF-B exists anymore.

All IOF values calculated in the C-HC brains can physiologically be correlated with the disruption of the B-CSF-B in C-HC at the level of the aqueduct and the lateral and the IVth ventricles. These calculated IOF values indicate the predominance of the disturbed CSF flow mechanism. The disturbance of the input mechanism is predominant if IOF > 1, while that of the output mechanism is predominant if IOF < 1.

Increases in the fractional blood volumes in the rat brain cortex of up to 160 times relative to the control values were detected using this CM of the DCE-MRI rat brain data. These changes might be physiologically correlated to the shrinkage of the brain tissue in the C-HC.

In the CSF pool, values of the relative volume of the CSF compartment less than 100% were estimated [13]. The blood compartment was detected in the lateral and the IVth ventricles and the values of its fractional volume ranged from 9.4 to 16.5% [13]. The largest values of the fractional volume of the blood compartment calculated in the normal brain, show the presence of the blood compartment in the small CSF spaces (a few pixels), due to the partial volume and noise effects affecting the MRI data in these regions. These data show that more accurate information could be extracted from the mathematically modeled DCE-MRI data acquired with higher spatial resolution and signal-to-noise ratio.

## 4. Conclusions

Compartment modeling of the DCE-MRI data provides quantitative information on the permeability of the BBB and B-CSF-B. Transfer rates as small as 0.0001 1/min were estimated by mathematically modeling the DCE-MRI data in the rat brain tissue pool [13]. The transfer rates ranged from 0 to 0.0005 1/min, showing no BBB breakdown in the rat brain affected by C-HC [13].

Tissue perfusion and kinetics of ^1^H nuclei in water molecules in different phases through different tissue and CSF compartments of the normal and disease-affected brain can be quantitatively evaluated *in vivo* using CM of the DCE-MRI data [13].

Statistical experimental analyses of these CMs for refined stages of disease severity are required for accurate description of the normal and/or of brain disease mechanisms in general and in C-HC in particular.

Even without any statistical analysis, the calculated IOF values for each CSF space analyzed clearly show that this is the most sensitive indicator of the CSF flow disturbances through the CSF spaces in C-HC. The predominance of the input or output flow disturbance in a CSF space at a disease stage can also be established based on the IOF values calculated.

CM of the DCE-MRI data may represent an important clinical imaging analysis method. It can provide quantitative information that can be used to assess physiology of the normal brain, mechanisms of brain diseases, including hydrocephalus, or responses to therapies.

## Appendices

### A. Tissue Concentration Modeling

Concentration of ^1^H spins in water molecules in the tissue can be estimated quantitatively by fitting the DCE-MRI data to the tissue concentration curve obtained by solving (4) and (5). Solution of (4) is given below

(A.1) $$\begin{array}{cc} C_{\text{EES}}\left(t\right) & =\frac{C_{\text{IN}}k_{\text{TRANS}}}{{\left(k_{\mathrm{EP}}-k_{\text{OUT}}\right)}^{2}}\\ & \;\times \left\{\left[\left(k_{\mathrm{EP}}-k_{\text{OUT}}\right)t-1\right]e^{-k_{\text{OUT}}t}+e^{-k_{\mathrm{EP}}t}\right\}. \end{array}$$

The contrast agents in DCE-MRI are extracellular and only transport of ^1^H nuclei in water molecules takes place between the EES and the IES compartments. Based on these assumptions, concentration of ^1^H spins in the IES and EES compartments will be nearly the same

(A.2) $$C_{\text{IES}}\left(t\right)≅C_{\text{EES}}\left(t\right).$$

The tissue concentration curves are then estimated by placing the *C*_EES_ and *C*_IES_ expressions above in (3).

### B. Concentration Curves

Kinetics of ^1^H spins in water molecules in the three pools evaluated can be obtained by fitting the concentration curves to (1), (7), (A.1). The concentration curves can be obtained by normalizing the MRI signal intensity, SI(*t*), over time to the signal intensity acquired before injecting the contrast agent, SI(0). The signal intensity at any of the time points considered is acquired using gradient echo with variable flip angle based pulse sequences with as short as possible echo times [13]:

(B.1) $$\frac{\text{SI}\left(0\right)}{\text{SI}\left(t\right)}=\frac{\left(1-e^{-T_{R}/T_{10}}\right)\left(1-e^{-T_{R}/T_{1}}\text{cosFA}\right)}{\left(1-e^{-T_{R}/T_{1}}\right)\left(1-e^{-T_{R}/T_{10}}\text{cosFA}\right)}.$$

Concentration is direct proportional to *T*_1_ [8, 9] and, if *T*_10_ is known, the concentration curves are obtained from (B.1). *T*_10_ and *T*_1_ represent the spin-lattice relaxation times of ^1^H nuclei in water molecules in a region evaluated (i.e., blood, tissue, or CSF) before and after infusion of the contrast agent. FA and *T*_*R*_ are pulse sequence parameters: the flip angle and the repetition time

(B.2) $$T_{1}=\frac{T_{R}}{\ln\left[\frac{\text{cosFA}-\left(\text{SI}\left(0\right)/\text{SI}\left(t\right)\right)\times \left(1-e^{-T_{R}/T_{10}}\text{cosFA}\right)/\left(1-e^{-T_{R}/T_{10}}\right)}{1-\left(\text{SI}\left(0\right)/\text{SI}\left(t\right)\right)\times \left(1-e^{-T_{R}/T_{10}}\text{cosFA}\right)/\left(1-e^{-T_{R}/T_{10}}\right)}\right].}$$

Initial *T*_10_ values of ^1^H nuclei in water molecules in the blood, tissue and CSF regions can be calculated *in vivo* [15]. Calculation of *T*_1_ is not necessary. The concentration curves in the blood data can be estimated by fitting the MRI data in the sagittal sinus [16] to (B.2).
